# The association between diet quality index-international and inflammatory markers in Iranian overweight and obese women

**DOI:** 10.3389/fnut.2023.1164281

**Published:** 2023-05-19

**Authors:** Farideh Shiraseb, Sara Ebrahimi, Sahar Noori, Reza Bagheri, Stacey Alvarez-Alvarado, Alexei Wong, Khadijeh Mirzaei

**Affiliations:** ^1^Department of Community Nutrition, School of Nutritional Sciences and Dietetics, Tehran University of Medical Sciences (TUMS), Tehran, Iran; ^2^The Ritchie Centre, Hudson Institute of Medical Research, Clayton, VIC, Australia; ^3^Department of Nutrition, Science and Research Branch, Islamic Azad University, Tehran, Iran; ^4^Department of Exercise Physiology, University of Isfahan, Isfahan, Iran; ^5^Department of Neurology, College of Medicine- Jacksonville, University of Florida, Jacksonville, FL, United States; ^6^Department of Health and Human Performance, Marymount University, Arlington, VA, United States; ^7^Food Microbiology Research Center, Tehran University of Medical Sciences, Tehran, Iran

**Keywords:** inflammatory markers, obese, overweight, women, diet quality index-international (DQI-I)

## Abstract

**Objectives:**

The present study was conducted to evaluate whether there is a link between the diet quality index (DQI) and markers of systemic inflammation in Iranian overweight and obese women.

**Methods:**

This cross-sectional study included 200 Iranian overweight and obese women aged 18–48 years. The DQI-international (DQI-I) comprises four main components: variety, adequacy, moderation, and overall balance. Blood samples were collected in a fasted state to measure inflammatory markers.

**Results:**

After adjusting for age, body mass index (BMI), physical activity, total energy intake, economic status, education, supplement intake, age of starting obesity, and history of body mass loss, a marginally significant negative association was observed between the homeostasis model assessment of insulin resistance (HOMA–IR) and the DQI–I (β: −0.015, 95% CI: −0.03, 0.000; *p* = 0.061). The results after adjustment showed that DQI–I has a negative association with high-sensitivity C-reactive protein (hs–CRP) concentrations (β: −0.031, 95% CI: −0.104, −0.031; *p* = 0.023). Furthermore, negative associations were observed between the adequacy component and levels of HOMA–IR (β: −0.025, 95% CI: −0.100, 0.047, *p* = 0.050) and hs-CRP (β: −0.615, 95% CI: −1.191, −0.020; *p* = 0.045). In addition, negative associations were found between transforming growth factor-β (TGF-β) and balance score (β: −6.270, 95% CI: −39.211, −3.661, *p* = 0.020), as well as HOMA–IR (β: −0.080, 95% CI: −0.202, −0.000, *p* = 0.041) and chemoattractant protein−1 (MCP−1) (β: −0.562, 95% CI: −11.414, −0.282, *p* = 0.021), with the various component. A marginally significant negative association between galectin 3 (Gal-3) and moderation score (β: −0.451, 95% CI: −1.171, 0.060, *p* = 0.060) was found. In addition, a marginally significant inverse association was also established between hs–CRP and variety score (β: −0.311, 95% CI: −0.970, 0.001, *p* = 0.052). The Receiver Operating characteristic (ROC) curve analysis demonstrated that DQI–I might better predict HOMA–IR with a cut point of 3.13 (AUC = 0.698, 0.511–0.699, *p* = 0.050).

**Conclusion:**

These findings showed that a higher adherence to diet quality and its components could probably be related to lowering the inflammatory markers considerably in overweight and obese women.

## Introduction

Recent evidence shows that more than 22% of Iranian adults were overweight and obese in 2018 (1). The prevalence of obesity in Iranian women is almost 57% higher than that in men (2). Obesity causes mild inflammation and can hasten the development of chronic diseases and their complications by secreting pro-inflammatory cytokines such as interleukin-6 (IL-6), tumor necrosis factor (TNF), and C-reactive protein (CRP) (3, 4). According to the literature, a low-grade inflammation arising from adiposity (5) also elevates some inflammatory factors, which are described below. White adipose tissue (WAT) secretes chemoattractant protein-1 (MCP-1) (6–8), which is a primary ligand for the chemokine receptor-2 (CCR2) (9), and high-sensitivity C-reactive protein (hs-CRP), which is a strong predictor of cardiovascular incidence (9, 10). CRP is an acute inflammatory protein that increases during inflammation and is used as an indicator of infection (11–13). Transforming growth factor-β (TGF-β) is one of the main adipokines involved in regulating inflammatory processes (14). The homeostasis model assessment of insulin resistance (HOMA-IR) indicates insulin resistance (IR), which increases in obese individuals (15, 16). TNF is an important mediator of insulin resistance in obesity and diabetes, through its capacity to diminish the tyrosine kinase activity of the insulin receptor, resulting in higher levels of inflammation (17–19). The plasminogen activator inhibitor-1 (PAI-1) is an inhibitor of tissue-type plasminogen activator (t-PA), synthesized by human adipose tissue and could play a role in inflammation and associated with IR parameters, including body mass index (BMI) and visceral fat (20–24). Pro-inflammatory cytokine interleukin-1 (IL-1) is a prototypical inflammatory cytokine that increases in response to higher cytotoxic IL-1β (25). Galectin-3 (Gal-3), in particular, plays both pro-inflammatory and anti-inflammatory roles, depending on the inflammatory status and the target cell or tissue (26, 27).

Diet plays a vital role in immune response control (28). Indeed, well-established evidence shows that malnutrition causes immunosuppression and inflammation (29). Some studies showed associations between diet quality and specific inflammatory markers such as hs-CRP (30, 31). Fung et al. examined associations between diet quality indices and plasma concentrations of hs-CRP, IL-6, E-selectin, soluble intercellular cell adhesion molecule 1, and soluble vascular cell adhesion molecule. The results revealed that the healthy eating index (HEI) and dietary quality index revised (DQI-R) scores were not significantly associated with any of the measured markers, while the Alternative Healthy Eating Index (AHEI) and alternative Mediterranean Diet (aMED) scores were significantly associated with lower concentrations of most of the measured markers (32). In addition, Kuczmarski et al. showed a 10% increase in the mean adequacy ratio (MAR) was significantly associated with lower hs-CRP (31). However, these diet quality indices have limitations, including a lack of components such as variety, adequacy, moderation, and overall balance. Therefore, evaluating other diet quality indices may be of importance to establish the relationship between diet quality and inflammatory markers in obesity (33–35).

The diet quality index-international (DQI-I) was developed according to international dietary recommendations to compare diet quality between different populations (36–38). The DQI-I evaluates the adequacy, moderation, variety, and balance aspects of a diet. It is useful to evaluate the undernutrition and overnutrition status in Iran, a country experiencing a nutritional transition over the past three decades (39, 40). The applicability of DQI-I in the Iranian population has been demonstrated in a previous study (41). However, no study has examined the associations between DQI-I and inflammatory markers in the Iranian population.

Given obesity is more prevalent among Iranian women and no evidence is available on the possible relationships between the DQI-I and inflammatory markers, this study aimed to examine associations between the DQI-I and inflammatory markers, including MCP-1, hs-CRP, IL-1β, Gal-3, HOMA-IR, TGF-β, and PAI-1, in Iranian overweight and obese women. The hypothesis is that DQI-I would be associated with inflammatory markers.

## Methods

### Study population

Two hundred pre-menopausal healthy women with overweight and obesity from Tehran, Iran, were included in this study. The inclusion criteria were as follows: (1) BMI in the range of 25–40 kg.m^−2^, (2) age range between 18 and 48 years, and (3) no history of chronic diseases (other than obesity). The exclusion criteria were medication usage, pregnancy, lactation, menopause, history of chronic disease or eating disorders, smoking, significant body mass fluctuations over the past year, and following any dietary regimen. Furthermore, individuals who did not answer more than 70 questions of the food frequency questionnaire (FFQ) and those with an energy intake under 800 and over 4,200 kcal/d were excluded (41). All participants signed an informed consent form prior to participation. This study was approved by the Ethics Committee of Tehran University of Medical Sciences (TUMS) (IR.TUMS.VCR.REC.1396.2615).

### Study design

The study had a cross-sectional design. Participant screening and data collection were completed between September 2018 and December 2018. A random cluster sampling method was used to recruit participants from health centers affiliated with TUMS. Data collection was completed in the Nutrition and Biochemistry Laboratory of the School of Nutrition and Dietetics at TUMS. All data were collected in one visit.

### Body composition and anthropometric measurements

The multi-frequency bioelectrical impedance analyzer (BIA), InBody 770 scanner, was used to measure body composition, including body mass, BMI, fat mass (FM), fat-free mass (FFM), fat mass index (FMI), and fat-free mass index (FFMI) (Inbody Co., Seoul, Korea). Participants were asked to urinate 30 min before the test. In addition, they fasted for 12 h and avoided any strenuous physical activity 48 h before the test. Height was measured using a digital Seca scale (Hamburg, Germany), with a precision of 0.5 cm. Waist circumference (WC), hip circumference (HC), and neck circumference (NC) were measured by a trained dietitian based on standard procedures (42). Waist-to-hip ratio (WHR) was calculated using the BIA, and waist-to-height ratio (WHtR) was calculated by dividing WC by (cm) height (cm).

### Biochemical parameters

Venous blood samples were obtained by a trained phlebotomist in the morning (8–10 a.m.), after 10–12 h of fasting. The serum was extracted and frozen at −80°C after separation for further analyses. A colorimetric approach based on the glucose oxidase-phenol amino phenazone (GOD-PAP) method was used to determine fasting blood glucose (FBG) levels. Triglyceride (TG) was evaluated using glycerine phosphate oxidase peroxidase (GPO-PAP). Low-density lipoprotein (LDL) was measured using the direct method. High-density lipoprotein cholesterol (HDL) and total cholesterol levels were calculated using an immune inhibition assay. The Pars Azmoon package (Pars Azmoon Inc. Tehran, Iran) was used for these analyses. An enzyme-linked immunosorbent assay (ELISA) approach was used to evaluate serum insulin levels (Human insulin ELISA kit, Monobind Inc., Lake Forest, United States). Insulin resistance was assessed by HOMA-IR, and the index was calculated using the following formula: HOMA-IR= (fasting plasma glucose (mmol/l) × fasting plasma insulin mIU/l)/22.5 (43). The concentrations of inflammatory markers such as hs-CRP and IL-1β were measured using the ELISA method. The active form of Gal-3 was also measured using the ELISA-Quantikine kits (R&D Systems, Minneapolis, MN). The ELISA approach was further used to test serum MCP-1 levels using a suitable kit (Zell Bio GmbH, ULM, Germany, assay range: 5 ng/L−1,500 ng/L, sensitivity: 2.4 ng/L). In triplicate, TGF-β (HUMAN TGF-BETA 1 Quantikine ELIZA kit R&D System-United States) and PAI-1 (Human PAI-1^*^96 T ELIZA kit Crystal Company) were assessed. The inter-assay and intra-assay variability for all biochemical tests were < 12% and 10%, respectively.

### Blood pressure

A physician used a mercury sphygmomanometer to measure systolic and diastolic blood pressure (SBP, DBP), following 15 min of rest (seated). Three measurements at 1-min interval were recorded, and the average of measurements was calculated.

### Dietary assessment

A dietary assessment was performed by a trained dietitian, using a semi-quantitative FFQ with 147 food items. The validity and reliability of instruments have been confirmed in previous studies (44, 45). Participants reported the frequency of consumption for a given serving of each food item during the last year on a daily, weekly, monthly, or yearly basis. Portion size for the consumed food was converted to grams using household measurements (46) and dietary data were analyzed using the Nutritionist IV software (version 7.0; N-Squared Computing, Salem, OR) (47).

### DQI-I calculation

The DQI-I comprises four main components, such as variety, adequacy, moderation, and balance. The score range of variety is between 0 and 20, which includes food group variety and protein source variety. The adequacy component consists of fruits, vegetables, and grains with a score range between 0 and 40. Total fat, saturated fat, cholesterol, sodium, and empty calorie foods make the moderation components with a score range between 0 and 30. The balance component includes macronutrient ratio and fatty acid ratio with a score range between 0 and 10. The total score range is between 0 and 100, and higher DQI-I scores indicate higher diet quality (48). Participants were classified into two groups, according to DQI-I scores median (49), lower diet quality ( ≤ 64), and higher diet quality (>64).

### Assessment of other variables

The characteristics of participants including age, marital status, and economic status were collected by a trained interviewer through a demographic questionnaire. To determine physical activity standards, the validated International Physical Activity Questionnaire (IPAQ) was calculated as minutes per week, according to metabolic equivalents (MET-min/week) (50, 51).

### Statistical analyses

All analyses were performed using a Statistical Package for Social Science (version 26.0; SPSS Inc., Chicago IL, United States). *P*- < 0.05 was considered statistically significant, and *P*-value of 0.06 was considered marginally significant. Mean and standard deviation (SD) were reported for continuous variables while the number and percentage were reported for categorical variables. Quantitative independent normality was tested using Kolmogorov–Smirnov's test (*p* > 0.05). In the crude model, the independent one-way analysis of variance (ANOVA) and chi-square (χ2) test were used to analyze continuous and categorical variables, respectively. Analysis of covariance (ANCOVA) was applied to control confounders, including age, physical activity, BMI, and total energy intake. Linear regression analysis was used to examine the association between DQI-I and inflammatory markers, including MCP-1, hs-CRP, PAI-1, Gal-3, IL-1β, TGF-β, and HOMA-IR. All assumptions of linear regression tests such as normality, normality of residual error, linearity, homoscedasticity, and collinearity were assessed. The analysis was adjusted for age, physical activity, BMI, and total energy intake. ROC curves were plotted with sensitivity (true–positive fraction) on the y-axis and 1-specificity (false–positive fraction) on the x-axis. The ROC curve was plotted for DQI-I value to inflammatory markers.

## Results

### Study population characteristics

A total of 200 Iranian overweight and obese women were included in the analysis. The majority of participants were married (75.3%), had a low level of education (52.7%), and had low and moderate socioeconomic status (74.2%). The mean age and BMI were 35.0 years (8.79) and 30.5 (4.12) kg.m^−2^, respectively. The mean of WHtR, FFM, and FM of individuals was 0.601 (0.000), 46.2 (4.26) kg, and 33.25 (5.27) kg, respectively. The mean DQI-I score was 64.5 (3.25).

### General characteristics of participants over DQI-I categories

The median of DQI-I indices was used to allocate participants into low and high DQI-I categories. Sociodemographic characteristics, anthropometrics indices, biochemical assessment, and blood pressure of participants before and after adjustment for cofounders are presented in Table 1. After adjustment for age, physical activity, and total energy intake, a marginally significant mean difference in BMI was found between medians of DQI-I (*p* = 0.061). No statistically significant difference was observed in the mean of biochemical parameters, body composition, anthropometric, and categorical variables between DQI-I medians in the crude and adjusted model (*p*>0.05).

**Table 1:** Characteristics of participants over medians of DQI-I (*n* = 200).

**Variables**	**DQI-I**		***P*-value**	***P*-value^†^**
	**Low**	**High**		
	**N**=**131 (** ≤ **64)**	**N**=**69 (**>**65)**		
Age (years)	36.5 ± 8.13	35.1 ± 9.32	0.275	0.141
Physical activity (MET-minutes/week)	1,134.4 ± 12.68	952.7 ± 1,949.46	0.443	0.475
Age stat obesity (years)	23.47 ± 8.464	21.743 ± 9.138	0.319	0.322
Anthropometric variables				
Weight (kg)	79.6 ± 11.07	78.5 ± 10.53	0.512	0.210
Height (cm)	161.3 ± 6.34	160.6 ± 5.80	0.442	0.422
HC (cm)	105.7 ± 6.18	105.0 ± 6.02	0.845	0.180
WC (cm)	97.9 ± 9.41	97.7 ± 9.31	0.921	0.836
NC (cm)	38.3 ± 9.82	36.2 ± 2.17	0.231	0.190
BMI (kg/m^2^)	30.6 ± 3.74	30.1 ± 3.99	0.900	0.061
Body composition				
WHR	0.9 ± 0.05	0.9 ± 0.05	0.520	0.415
WHtR	0.6 ± 0.05	0.6 ± 0.05	0.841	0.091
FM (kg)	33.8 ± 8.75	33.4 ± 8.30	0.754	0.696
FFM (kg)	46.8 ± 5.88	45.6 ± 5.36	0.161	0.302
FFMI	17.9 ± 1.46	17.6 ± 1.52	0.165	0.420
FMI	13.0 ± 3.34	13.0 ± 3.32	0.995	0.194
Biochemical components			
TG (mg/dl)	121.3 ± 60.78	117.7 ± 62.53	0.710	0.456
TC (mg/dl)	180.3 ± 31.08	181.9 ± 33.43	0.751	0.611
HDL-c (mg/dl)	47.2 ± 8.96	47.0 ± 9.98	0.922	0.667
LDL-c (mg/dl)	97.4 ± 21.70	99.6 ± 22.73	0.521	0.935
FBS (mg/dl)	86.6 ± 9.72	87.4 ± 8.87	0.570	0.661
Insulin (mIU/ml)	1.2 ± 0.23	1.2 ± 0.22	0.902	0.811
GOT (μKat/L)	18.2 ± 8.55	17.8 ± 6.60	0.740	0.482
GPT (μKat/L)	20.1 ± 15.03	19.7 ± 12.04	0.862	0.610
SBP (mmHg)	111.0 ± 13.65	110.3 ± 12.48	0.710	0.875
DBP (mmHg)	77.3 ± 9.38	77.3 ± 8.96	0.995	0.655
Categorical variables				
Economic status			0.841	0.691
Low level	31 (63.3)	18 (36.7)		
Moderate level	64 (67.4)	31 (32.6)		
High level	33 (63.5)	19 (36.5)		
Education level			0.960	0.991
Illiterate	1 (50)	1 (50)		
Under diploma	19 (67.9)	9 (32.1)		
Diploma	50 (66.7)	25 (33.3)		
Master and higher	61 (64.2)	34 (35.8)		
Marital status			0.463	0.452
Single	30 (61.2)	19 (38.8)		
Married	101 (66.9)	50 (33.1)		
Supplement intake				
Yes	43 (62.4)	24 (35.8)	0.410	0.796
No	29 (60.4)	19 (39.6)		
Losing weight history				
Yes	46 (69.7)	20 (30.3)	0.06	0.087
No	26 (54.2)	22 (45.8)		

DQI-I, diet quality index-international; MET, metabolic equivalents; HP, hip circumference; WC, waist circumference; NC, neck circumference; BMI, body mass index; WHR, waist-to-hip ratio; WHtR, waist-to-height ratio; FM, fat mass; FFM, fat-free mass; FFMI, fat-free mass index; FMI, fat mass index; TG, triglycerides; HDL, high-density lipoprotein; LDL, low-density lipoprotein; FBS, fasting blood sugar.

Quantitative variables were reported as mean±SD and categorical variables with numbers and percentages in parenthesis.

P-values resulted from the analysis of one-way ANOVA for continuous variables and chi-square test for categorical variables.

^†^Adjusted p-value obtained from an analysis of covariance test after adjusting for age, physical activity, total energy intake, and BMI. BMI was considered as collinear variable for anthropometric and body composition variables.

P < 0.05 was considered significant, and p-values of 0.06 and 0.07 were considered marginally significant.

### Dietary intake of study population over DQI-I categories

Dietary intake according to the DQI-I median (low and high DQI-I categories) is presented in Table 2. No statistically significant difference was observed in the mean of energy intake between low and high DQI-I categories [2,517 [738] and 2,649 [676], respectively] (*p* =0.150). All variables adjusted for confounding variables including age, BMI, physical activity, and total energy intake. The intake of fruits, vegetables, legumes, and low-fat dairy products was higher in participants with a higher DQI-I score (*p* ≤ 0.05). The intake of high-fat dairy products, red meat, and fast foods was significantly lower in participants with higher DQI-I scores (*p* ≤ 0.05), after adjustment for confounding variables. The intake of carbohydrates, total dietary fiber, iron, selenium, copper, calcium, magnesium, potassium, manganese, vitamin C, vitamin K, B6, B9, thiamin, and biotin was significantly higher in participants with higher DQI-I compared to participants with lower DQI-I (*p* ≤ 0.05). Furthermore, the intake of fat, MUFA, PUFA, SFA, linolenic acid, linoleic acid, vitamin E, and vitamin B12 was significantly lower in participants with higher DQI-I (*p* < 0.05).

**Table 2:** Dietary intakes over the categories of DQI-I (*n* = 200).

**Variables**	**Median of DQI**		**p-value**	**p-value^†^**
	**Low (** < =**64)**	**High (**>**65)**		
**Food groups**				
Cereal (g/d)	120.8 ± 14.32	60.5 ± 14.48	**0.004**	**0.010**
Whole grain (g/d)	60.3 ± 6.04	68.4 ± 6.11	**0.022**	0.075
Refined grain (g/d)	334.9 ± 19.36	389.3 ± 19.58	**0.025**	**0.050**
Fruits (g/d)	397.8 ± 29.30	590.2 ± 29.63	**< 0.001**	**< 0.001**
Vegetables (g/d)	323.0 ± 21.94	466.2 ± 22.19	**< 0.001**	**< 0.001**
Starchy vegetables	44.6 ± 3.58	46.7 ± 3.62	0.915	0.686
Legumes (g/d)	40.9 ± 4.33	57.8 ± 4.38	**0.020**	**0.007**
Nuts (g/d)	15.6 ± 1.52	14.0 ± 1.54	0.651	0.462
Dairy low fat (g/d)	244.9 ± 20.82	347.4 ± 21.05	**0.001**	**0.001**
Dairy high fat (g/d)	120.8 ± 14.32	60.5 ± 14.48	**0.003**	**0.004**
Eggs (g/d)	20.3 ± 1.28	21.6 ± 1.29	0.942	0.491
White Meat (g/d)	48.5 ± 4.94	46.6 ± 4.99	0.825	0.798
Red meat (g/d)	38.9 ± 1.66	33.3 ± 1.68	0.726	**0.024**
Sweets deserts (g/d)	73.2 ± 7.34	72.5 ± 7.42	0.687	0.955
Salty snack (g/d)	36.8 ± 4.12	37.0 ± 4.17	0.489	0.975
Fast foods (g/d)	45.1 ± 2.89	37.0 ± 2.89	0.670	**0.042**
**Energy and macronutrients**				
Energy (kcal/d)	2,517.1 ± 738.43	2,649.0 ± 676.06	0.150	-
Carbohydrates (g/d)	338.7 ± 4.06	395.5 ± 4.08	**< 0.001**	**< 0.001**
Fat (g/d)	105.0 ± 1.75	80.5 ± 1.76	**< 0.001**	**< 0.001**
Protein (g/d)	86.8 ± 1.82	91.1 ± 1.83	**0.012**	0.101
**Micronutrients and total fiber**				
Total fiber (g/d)	38.7 ± 1.30	51.4 ± 1.31	**< 0.001**	**< 0.001**
MUFA (g/d)	35.8 ± 0.78	26.1 ± 0.78	**< 0.001**	**< 0.001**
PUFA (g/d)	22.0 ± 0.78	17.8 ± 0.78	**0.030**	**< 0.001**
SFA (g/d)	32.6 ± 0.66	22.4 ± 0.66	**< 0.001**	**< 0.001**
Trans fat (g/d)	0.0 ± 0.00	0.0 ± 0.00	0.934	0.270
Linolenic acid (g/d)	1.3 ± 0.05	1.0 ± 0.05	**0.025**	**0.003**
Linoleic acid (g/d)	19.2 ± 0.76	15.2 ± 0.77	**0.025**	**< 0.001**
EPA (g/d)	0.0 ± 0.00	0.0 ± 0.00	0.621	0.990
DHA (g/d)	0.1 ± 0.01	0.1 ± 0.01	0.680	0.970
Iron (mg/d)	17.4 ± 0.26	19.9 ± 0.26	**< 0.001**	**< 0.001**
Zinc (mg/d)	12.6 ± 0.22	13.1 ± 0.22	**0.011**	0.175
Selenium (mcg/d)	115.8 ± 3.00	125.1 ± 3.02	**0.004**	**0.031**
Copper (mg/d)	1.8 ± 0.04	2.1 ± 0.04	**< 0.001**	**< 0.001**
Calcium (mg/d)	1,102.9 ± 31.09	1,217.6 ± 31.27	**0.014**	**0.012**
Magnesium (mg/d)	6.6 ± 0.18	7.3 ± 0.18	**< 0.001**	**< 0.001**
Potassium (mEq/d)	3,928.9 ± 97.91	4,665.7 ± 98.47	**< 0.001**	**< 0.001**
Manganese (mg/d)	429.3 ± 7.50	488.1 ± 7.54	**0.007**	**0.014**
Sodium (mg/d)	4,226.9 ± 118.35	4,249.1 ± 119.02	0.427	0.895
Chromium (mg/d)	0.1 ± 0.00	0.1 ± 0.00	0.031	0.090
Vitamin C (mg/d)	156.9 ± 9.66	217.1 ± 9.72	**< 0.001**	**< 0.001**
Vitamin E (mg/d)	18.6 ± 0.87	15.8 ± 0.88	0.284	**0.020**
Vitamin A (mg/d)	783.7 ± 34.53	770.4 ± 34.72	0.070	0.781
Vitamin K (mg/d)	193.7 ± 15.49	236.8 ± 15.57	**0.003**	**0.050**
Vitamin D (mg/d)	1.8 ± 0.17	2.1 ± 0.17	0.210	0.330
Thiamin (mg/d)	1.9 ± 0.03	2.1 ± 0.03	**< 0.001**	**< 0.001**
Riboflavin (mg/d)	2.1 ± 0.05	2.2 ± 0.05	**0.020**	0.240
Niacin (mg/d)	24.5 ± 0.67	26.1 ± 0.67	**0.003**	0.102
Biotin (mg/d)	35.2 ± 1.61	41.4 ± 1.62	**< 0.001**	**0.008**
Vitamin B6 (mg/d)	2.0 ± 0.04	2.2 ± 0.04	**0.001**	**0.001**
Folate (mcg/d)	556.1 ± 9.76	715.3 ± 14.04	**< 0.001**	**< 0.001**
Vitamin B12 (mcg/d)	4.6 ± 0.20	4.0 ± 0.20	0.891	**0.042**

P < 0.05 was considered significant, and p-values of 0.06 and 0.07 were considered marginally significant.

MUFA, monounsaturated fatty acid; PUFA, polyunsaturated fatty acid; SFA, saturated fatty acid; EPA, eicosapentaenoic acid; DHA, docosahexaenoic acid.

Mean ± SD from ANOVA is presented.

Nutrients and food groups were adjusted for age, BMI, physical activity, and total energy intake.

^**†**^Adjusted p-value obtained from an analysis of covariance after adjustment for energy intake.

Bold values indicate significance.

### Inflammatory markers across the low and high categories of DQI-I and its components

No statistically significant difference was found in the level of PAI-1, Gal-3, and MCP-1 between the low and high DQI-I categories, before adjustment for cofounders (*p*>0.05). However, after adjustment for age, BMI, physical activity, total energy intake, socioeconomic status, education, supplement intake, age of starting obesity, and history of weight loss, the mean of PAI-1, Gal-3, hs-CRP, and MCP-1 was lower in participants with higher DQI-I (*p* < 0.05; Table 3). HOMA-IR was significantly lower in women with a higher score of adequacy (*p* < 0.05), both in the crude model and two adjusted models (adjusted for age, BMI, physical activity, total energy intake, economic status, and education in Model 1; and further adjustment for supplement intake, age of starting obesity, and history of weight loss in Model 2). Lower levels of PAI-1 and TGF-β were found in participants with higher balance scores after controlling for age, BMI, physical activity, total energy intake, economic status, education, supplement intake, age of starting obesity, and history of weight loss (*p* = 0.042, *p* = 0.040, respectively). Furthermore, the levels of IL-1β (*p* = 0.011) in Model 1 and MCP-1(*p* = 0.031) in Model 2 were lower in participants with a higher moderation score. In addition, hs-CRP was lower in participants with a higher moderation score (Table 3).

**Table 3:** Inflammatory markers over the categories of DQI-I and its components (*n* = 200).

**Variables**		**DQI–I**		***P*-value^†^**	**Adequacy**		***P*-value^†^**	**Balance**		**P–value^†^**	**Moderation**		**P-value^†^**	**Variety**		**P-value^†^**
		**Low**	**High**		**Low**	**High**		**Low**	**High**		**Low**	**High**		**Low**	**High**	
HOMA-IR	Crude	3.2 ± 1.31	3.3 ± 1.37	0.542	3.3 ± 0.20	1.9 ± 0.43	**0.006**	3.1 ± 0.22	2.6 ± 0.51	0.370	3.1 ± 0.23	2.6 ± 0.44	0.276	3.2 ± 0.40	2.9 ± 0.24	0.520
	Model1	2.9 ± 0.3	3.1 ± 0.41	0.6310	3.2 ± 0.24	1.7 ± 0.53	**0.028**	3.0 ± 0.26	2.5 ± 0.69	0.550	3.1 ± 0.25	2.3 ± 0.57	0.230	3.2 ± 0.53	2.8 ± 0.29	0.541
	Model2	3.2 ± 0.31	2.6 ± 0.44	0.341	3.3 ± 0.26	1.9 ± 0.57	**0.046**	3.1 ± 0.25	2.5 ± 0.66	0.475	3.0 ± 0.27	2.9 ± 0.58	0.952	3.0 ± 0.61	2.9 ± 0.30	0.870
MCP-1 (mg/L)	Crude	62.7 ± 111.01	37.1 ± 58.90	0.110	56.5 ± 18.50	52.3 ± 38.29	0.925	59.2 ± 18.14	37.7 ± 41.23	0.630	66.0 ± 18.43	18.2 ± 35.10	0.238	27.6 ± 31.56	66.1 ± 19.20	0.300
	Model1	72.5 ± 23.14	39.3 ± 31.38	0.430	59.5 ± 21.02	57.2 ± 45.36	0.964	69.9 ± 19.33	−4.1 ± 51.34	0.203	70.7 ± 19.38	43.2 ± 43.56	0.180	57.9 ± 41.08	59.4 ± 22.40	0.975
	Model2	88.4 ± 27.05	18.8 ± 22.01	**0.041**	63.3 ± 21.62	56.9 ± 47.24	0.912	71.5 ± 18.58	−5.4 ± 53.89	0.200	69.2 ± 19.37	32.9 ± 11.27	**0.031**	56.2 ± 45.98	63.8 ± 22.73	0.890
PAI-1 (mg/L)	Crude	19.9 ± 38.11	11.2 ± 17.77	0.175	17.9 ± 6.95	16.9 ± 14.38	0.940	19.1 ± 6.81	10.7 ± 15.48	0.625	21.0 ± 6.96	5.8 ± 13.26	0.316	6.7 ± 11.84	21.8 ± 7.20	0.281
	Model1	21.5 ± 9.47	14.0 ± 12.84	0.668	18.7 ± 8.36	19.2 ± 18.05	0.985	22.4 ± 7.78	−1.8 ± 20.65	0.290	23.1 ± 7.76	−0.8 ± 17.45	0.224	13.00 ± 16.2	20.9 ± 8.88	0.690
	Model2	28.7 ± 11.32	4.0 ± 15.91	**0.028**	21.1 ± 9.29	15.8 ± 20.30	0.820	22.9 ± 8.16	−0.4 ± 23.68	**0.042**	24.0 ± 8.04	0.8 ± 17.12	0.060	14.6 ± 19.75	21.7 ± 9.76	0.771
Gal-3 (mg/L)	Crude	5.4 ± 8.82	3.9 ± 7.14	0.517	3.6 ± 1.19	3.1 ± 2.48	0.850	3.8 ± 1.17	2.1 ± 2.66	0.551	4.3 ± 1.19	1.0 ± 2.27	0.200	1.5 ± 2.03	4.3 ± 1.24	0.250
	Model1	4.4 ± 1.56	2.8 ± 2.11	0.588	3.8 ± 1.40	3.5 ± 3.03	0.921	4.5 ± 1.29	−0.3 ± 3.44	0.210	4.6 ± 1.29	−0.0 ± 2.90	0.167	3.0 ± 2.74	4.0 ± 1.49	0.775
	Model2	5.5 ± 1.84	1.3 ± 2.59	**0.024**	4.2 ± 1.48	3.3 ± 3.24	0.822	4.6 ± 1.28	−0.3 ± 3.72	0.231	4.6 ± 1.30	1.3 ± 2.77	0.320	3.0 ± 3.15	4.3 ± 1.56	0.731
Hs-CRP (mg/l)	Crude	4.4 ± 5.06	4.6 ± 4.89	0.787	4.3 ± 0.86	3.5 ± 1.79	0.694	4.3 ± 0.85	3.2 ± 1.93	0.590	3.8 ± 0.87	5.4 ± 1.66	0.414	5.8 ± 1.46	3.5 ± 0.89	0.202
	Model1	4.2 ± 0.88	5.5 ± 1.20	0.420	4.7 ± 0.77	3.2 ± 1.66	0.425	4.5 ± 0.74	4.0 ± 1.97	0.822	3.8 ± 0.70	7.1 ± 1.57	0.093	7.1 ± 1.40	3.4 ± 0.76	0.042
	Model2	4.4 ± 0.88	5.1 ± 1.24	0.690	4.9 ± 0.85	3.5 ± 1.87	0.551	4.7 ± 0.77	3.6 ± 2.24	0.641	4.1 ± 0.72	7.2 ± 1.55	0.061	7.9 ± 1.66	3.5 ± 0.82	**0.043**
IL-1β (mg/L)	Crude	0.3 ± 0.56	0.4 ± 0.58	0.621	0.3 ± 0.10	0.3 ± 0.21	0.910	0.3 ± 0.10	0.3 ± 0.22	0.987	0.2 ± 0.10	0.6 ± 0.19	0.126	0.5 ± 0.16	0.2 ± 0.10	0.095
	Model1	0.3 ± 0.12	0.4 ± 0.16	0.640	0.3 ± 0.09	0.4 ± 0.20	0.550	0.3 ± 0.08	0.4 ± 0.23	0.560	0.2 ± 0.07	0.8 ± 0.17	0.011	0.6 ± 0.17	0.2 ± 0.09	0.016
	Model2	0.4 ± 0.12	0.2 ± 0.17	0.545	0.3 ± 1.87	0.5 ± 0.21	0.485	0.3 ± 0.08	0.5 ± 0.25	0.471	0.2 ± 0.09	0.7 ± 0.27	0.095	0.61 ± 0.2	0.3 ± 0.10	**0.050**
TGF-β (mg/L)	Crude	61.2 ± 18.55	55.8 ± 8.87	0.515	59.9 ± 4.42	52.9 ± 10.84	0.560	59.9 ± 4.42	52.9 ± 10.81	0.560	58.3 ± 4.46	62.6 ± 10.94	0.711	54.9 ± 5.22	64.1 ± 6.02	0.271
	Model1	57.7 ± 6.15	62.1 ± 8.14	0.710	66.2 ± 17.84	51.3 ± 20.60	0.714	64.9 ± 5.61	28.9 ± 22.53	0.261	58.0 ± 6.32	66.9 ± 22.59	0.752	66.2 ± 17.84	51.3 ± 20.67	0.711
	Model2	58.3 ± 12.24	61.1 ± 17.66	0.925	67.6 ± 25.74	49.8 ± 29.75	0.796	70.7 ± 5.50	26.3 ±−3.10	0.040	59.8 ± 10.58	56.8 ± 43.54	0.960	67.4 ± 25.74	49.8 ± 29.75	0.790

P < 0.05 was considered significant, and p-values of 0.06 and 0.07 were considered.

DQI-I, diet quality index-international; HOMA-IR, homeostasis model assessment of insulin resistance; MCP-1, chemoattractant protein-1; PAI-1, plasminogen activator inhibitor-1; Gal-3, galectin-3; hs-CRP, high-sensitivity C-reactive protein; IL-1β, interleukin-1beta; TGF-β, transforming growth factor-β.

Mean ± SD from ANOVA and mean ± SE from ANCOVA are presented.

Model 1: analysis was adjusted for age, BMI, physical activity, total energy intake, economic status, and education level.

Model 2: further adjusted for supplement intake and age of starting obesity and history of weight loss, marginally significant.

Bold values indicate significance.

### Association between inflammatory markers with DQI-I and its components

No significant association between HOMA-IR and DQI–I (β: −0.021, 95% CI: −0.040, 0.005, *p* = 0.101) was found in the crude model. However, in Model 2, a marginally significant association (β: −0.015, 95% CI: −0.030, 0.000, *p* = 0.061) was observed. In addition, in Model 2, a negative association between DQI–I and level of hs–CRP (β: −0.031, 95% CI: −0.104, −0.031, *p* = 0.023) was observed. Furthermore, there were negative associations between the adequacy component and the level of HOMA–IR (β: −0.025, 95% CI: −0.100, 0.047, *p* = 0.050) and hs–CRP (β: −0.615, 95% CI: −1.191, −0.020, *p* = 0.045), after adjusting for confounders (Model 2; Table 4). In addition, higher balance component scores were negatively associated with TGF–β (β: −6.270, 95% CI: −39.211, −3.661, *p* = 0.020) in Model 2. There was a negative and marginal association between higher moderation scores and Gal−3 (β: −0.451, 95% CI: −1.171,0.060, *p* = 0.060) (Model 2). In Model 2, a negative association was found between variety score and HOMA–IR (β: −0.080, 95% CI: −0.202, −0.000, *p* = 0.041), MCP−1 (β: −0.562, 95% CI: −11.414, −0.282, *p* = 0.021), and hs–CRP levels (β: −0.311, 95% CI: −0.970,0.001, *p* = 0.052) (Table 4).

**Table 4:** Association between DQI-I and its components and inflammatory markers (*n* = 200).

**Variables**		**DQI-I**		**P-value^†^**	**Adequacy**		**P-value^†^**	**Balance**		**P-value^†^**	**Moderation**		**P-value^†^**	**Variety**		**P-value^†^**
		β	**95%CI**		β	**95%CI**		β	**95%CI**		β	**95%CI**		β	**95%CI**
HOMA-IR	Crude	−0.021	−0.040,0.005	0.101	−0.051	−0.110,0.002	0.076	−0.031	−0.110,0.051	0.440	0.009	−0.030,0.051	0.680	0.008	−0.365,0.382	0.960
	Model1	−0.011	−0.040,0.002	0.203	−0.140	−0.281, −0.011	**0.034**	−0.012	−0.092,0.060	0.765	−0.010	−0.06,0.02	0.461	−0.011	−0.090,0.065	0.160
	Model2	−0.015	−0.030,0.000	0.061	−0.025	−0.100,0.047	**0.050**	−0.020	−0.110,0.061	0.590	−0.020	−0.071,0.020	0.404	−0.080	−0.202,−0.000	**0.041**
MCP-1 (mg/L)	Crude	1.302	−0.651,3.265	0.190	4.271	−0.071,8.621	**0.052**	−1.830	−7.891,4.230	0.551	1.410	−1.991,4.820	0.412	2.611	−5.051,10.292	0.505
	Model1	1.011	−1.212,3.243	0.360	0.433	−1.382, 2.265	0.621	−1.551	−8.382,5.280	0.651	0.520	−3.351,4.392	0.791	−1.553	−8.381,−5.280	0.153
	Model2	0.200	−1.092,1.500	0.221	−0.445	−3.080,2.200	0.722	0.850	−7.051,8.760	0.830	1.080	−3.330,5.490	0.620	−0.562	−11.414,−0.282	**0.021**
PAI-1 (mg/L)	Crude	0.464	−0.275,1.211	0.213	2.135	0.174,4.105	**0.031**	−0.440	−2.740,1.850	0.70	0.322	−0.964,1.601	0.620	1.610	−1.281,4.500	0.270
	Model1	0.154	−0.743,1.050	0.730	2.197	−0.421,4.800	0.092	−1.302	−4.031,1.410	0.340	0.041	−1.476,1.550	0.951	−1.300	−4.032,1.411	0.341
	Model2	0.181	−0.512, −0.210	0.241	2.212	−0.991,5.420	0.171	−0.340	−3.650,2.962	0.830	0.261	−1.472,2.010	0.760	0.951	−5.031,4.745	0.950
Galectin 3 (mg/L)	Crude	0.080	−0.221,0.390	0.590	0.560	−0.091,1.225	0.095	−0.124	−1.081,0.825	0.792	−0.129	−0.630,0.394	0.641	0.280	−0.881,1.440	0.630
	Model1	−0.051	−0.440,0.334	0.785	0.490	−0.324,1.310	0.220	−0.283	−1.455,0.884	0.625	−0.352	−0.971,0.274	0.260	−0.281	−1.451,0.880	0.621
	Model2	−0.114	−0.631,0.300	0.590	0.431	−0.540,1.410	0.374	−0.354	−1.725,1.025	0.600	−0.451	−1.171,0.060	0.060	0.195	−1.375,1.745	0.790
Hs-CRP (mg/l)	Crude	−0.010	−0.114,0.075	0.710	−0.065	−0.284,0.165	0.580	−0.037	−0.342,0.265	0.810	−0.011	−0.17,0.15	0.90	0.008	−0.361,0.380	0.961
	Model1	−0.041	−0.140,0.050	0.261	−0.264	−0.744,0.214	0.26	−0.094	−0.410,0.235	0.570	−0.052	−0.230,0.111	0.525	−0.091	−0.410,0.231	0.575
	Model2	−0.031	−0.104, −0.031	**0.023**	−0.615	−1.191, −0.020	**0.045**	−0.032	−0.340,0.405	0.860	−0.040	−0.232,0.150	0.680	−0.311	−0.970,0.001	**0.052**
IL1-b (mg/L)	Crude	0.000	−0.011,0.010	0.981	0.006	−0.016,0.034	0.605	−0.006	−0.041,0.020	0.714	−0.004	−0.021,0.01	0.651	0.011	−0.021,0.051	0.516
	Model1	−0.003	−0.17,0.001	0.661	0.001	−0.009,0.012	0.784	−0.011	−0.051,0.024	0.510	−0.009	−0.030,0.010	0.404	−0.012	−0.051,0.020	0.511
	Model2	−0.001	−0.015,0.205	0.711	0.010	−0.006,0.030	0.173	0.008	−0.035,0.051	0.710	−0.004	−0.020,0.010	0.769	0.008	−0.041,0.060	0.781
TGF-β (mg/L)	Crude	0.231	−0.650,1.131	0.571	1.280	−0.961,3.535	0.230	0.540	−3.144,4.230	0.751	−0.200	−1.864,1.462	0.790	0.542	−3.141,4.231	0.752
	Model1	−0.730	−3.730,2.261	0.490	−0.811	−14.041,12.4051	0.851	−3.781	−16.606,9.030	0.410	−1.645	−6.845,3.552	0.381	−3.781	−16.600,9.031	0.415
	Model2	−0.711	−16.440,15.005	0.660	−1.461	−72.138,69.203	0.830	−6.270	−39.211,−3.661	**0.020**	−1.051	−36.471,34.366	0.770	−7.350	−126.281,111.570	0.096

DQI-I, diet quality index-international; HOMA-IR, homeostasis model assessment of insulin resistance; MCP-1, chemoattractant protein-1; PAI-1, plasminogen activator inhibitor-1; GAL-3, galectin-3; hs-CRP, high-sensitivity C-reactive protein; IL-1β, interleukin-1beta; TGF-β, transforming growth factor-β.

Model 1: analysis was adjusted for age, BMI, physical activity, total energy intake, economic status, and education status.

Model 2: further adjusted for supplement intake and age of starting obesity and history of losing weight.

P < 0.05 was considered significant, and p-values of 0.06 and 0.07 were considered marginally significant.

Bold values indicate significance.

### Prediction of inflammatory markers by ROC curve analysis

**Figure 1:**
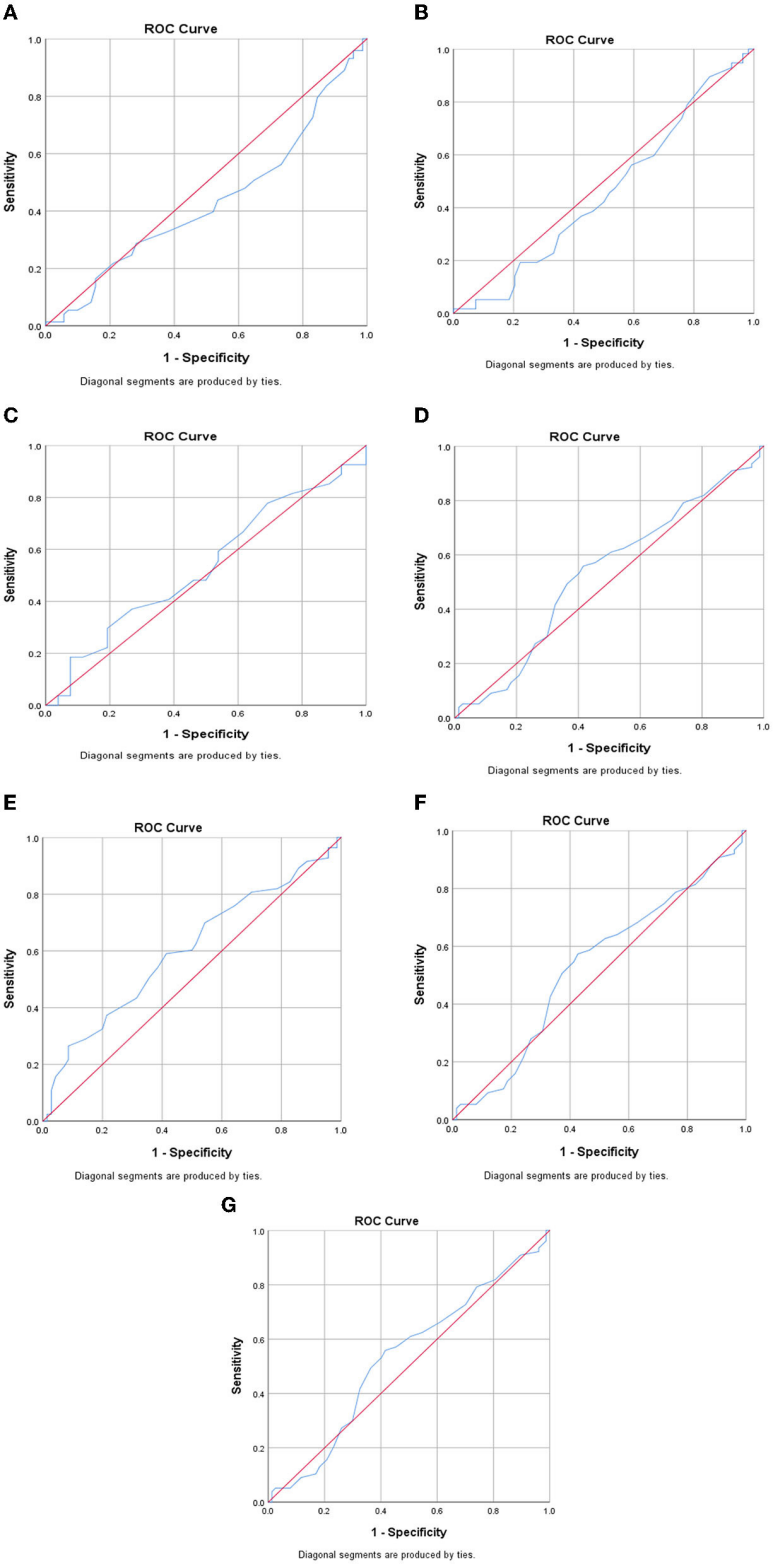
ROC curve of DQ-I to predict inflammatory markers. **(A)** MCP-1, **(B)** PAI-1, **(C)** Gal-3, **(D)** hs-CRP, **(E)** HOMA-IR, **(F)** IL-1β, **(G)** TGF-β.

Figure 1 demonstrates the ROC curve of DQI-I to predict inflammatory markers. This analysis showed the calculated area under the curve (AUC) with 95% CI for HOMA-IR (Figure 1E), with a cut point of 3.13 (AUC=0.698, 0.511–0.699, *p* = 0.05), which was statistically significant. Furthermore, the AUC with 95% CI for MCP-1 (Figure 1A) with a cut point of 15.4 (AUC=0.433, CI = 0.339–0.527), PAI-1 (Figure 1B) with a cut point of 6.21 (AUC=0.459 CI=0.350–0.567), Gal-3 (Figure 1C) with a cut point 1.23 (AUC = 0.533, CI = 0.376–0.691), hs-CRP (Figure 1D) with a cut point a 2.25 (AUC = 0.532 CI = 0.440-0.624), IL-1β (Figure 1F) with a cut point of 0.36 (AUC = 0.529, CI = 0.436–0.622), and TGF-β (Figure 1G) with a cut point of 65.06 (AUC = 0.522, CI = 0.401–0.528) were non-significant (*p*>0.05). These findings show that DQI-I might better predict HOMA-IR than the other inflammatory markers in this study population.

## Discussion

This study examined associations between inflammatory markers, including HOMA-IR, MCP-1, PAI-1, Gal-3, hs-CRP, IL-1β, TGF-β, and DQI-I in Iranian overweight and obese women. DQI-I was negatively associated with hs-CRP and HOMA-IR. Furthermore, there were negative associations between the adequacy score and hs-CRP and HOMA-IR, as well as between the balance score and TGF-β. In addition, the variety score was inversely associated with MCP-1, hs-CRP, and HOMA-IR, while the moderation score was negatively related to Gal-3 levels. These results are in line with our hypothesis that DQI-I is probably associated with inflammatory markers. Due to the other studies that showed the role of inflammation in obesity and causing related comorbidities and complications such as metabolic syndrome and cardiovascular disease, our findings are of significance (52–54).

The findings of this study showed that hs-CRP is negatively associated with total DQI-I score and score of adequacy and variety. The evidence shows that a healthy diet may be associated with lower levels of inflammation (55). The variety of components in the DQI-I emphasizes the higher intake of fruits and vegetables, meat, poultry, fish, egg, dairy, beans, and grains (35). In line with our findings, a previous study found an inverse link between prudent dietary patterns and hs-CRP levels (56). A prudent dietary pattern was characterized by a higher intake of fruit, vegetables, legumes, fish, poultry, and whole grains, which was similar to the food groups in the variety of components of our study. Furthermore, a higher intake of fruits and vegetables is associated with a lower level of inflammation, which might be attributed to anti-inflammatory properties of phytochemicals in fruits and vegetables (55). The adequacy component recommends a higher intake of fiber and vitamin C. Fruits and vegetables are also sources of fiber and vitamin C. Previous studies have shown inverse associations between fiber and vitamin C and hs-CRP (57–59). In addition, a previous study reported a negative association between the Mediterranean index and hs-CRP level (60). The Mediterranean diet was characterized by a higher intake of fruits, vegetables, beans, fish, and fiber, which was comparable to the adequacy component in this study that recommends a higher intake of fiber and vitamin C (32, 61, 62).

This study found a marginally significant association between DQI-I and HOMA-IR. In contrast with our findings, Alkerwi et al. recruited 1,352 participants, aged 18–69 years from Luxembourg, and found no association between the DQI-I and HOMA-IR (49). While the current study only included women, Alkerwi et al. investigated both women and men (49). On the other hand, the findings of our study showed an inverse association between the score of adequacy and lower HOMA-IR. The higher score of adequacy implies a higher intake of vegetables, fruit, grain, fiber, protein, iron, calcium, and vitamin C (35). The evidence shows that the higher consumption of fruits and vegetables is associated with a lower concentration of oxidative stress markers that trigger inflammatory pathways related to IR (63–65). This might explain the mechanism of the association between adequacy score and HOMA-IR.

The findings showed that a healthy diet such as the Mediterranean diet might have a relationship with a lower level of inflammation while unhealthy diets induce pro-inflammatory cytokines such as IL-1 (55, 66, 67). No investigation has examined the association between DQI-I and IL-1β; however, few studies investigated the association between IL-6 and diet quality (48, 56). The Multi-Ethnic Study of Atherosclerosis (MESA) study reported that a healthy dietary pattern was inversely associated with IL-6. Furthermore, this study showed a negative association between IL-6 and a higher intake of vegetables, fruit, whole grains, low-fat dairy products, and poultry (68). A Belgian epidemiological study reported that a dietary index, including dietary diversity, quality, and equilibrium, which was used to measure adherence to the Flemish food-based dietary guidelines, was inversely associated with IL-6 (69). Furthermore, prior research on individuals with metabolic syndrome (Mets) reported an inverse association between higher adherence to Mediterranean-style diet and IL-6, IL-7, and IL-18 (70).

The current findings demonstrated a negative association between the component variety and MCP-1. While there is limited information on the association between diet quality and MCP-1, previous studies reported that the consumption of a healthy diet such as the Mediterranean diet or a higher intake of fruits and vegetables and vitamin C is associated with a lower level of systematic inflammation (70, 71). Consistent with our findings, a study of 66 participants from Spain found a negative association between the Mediterranean diet and MCP-1, which could be due to antioxidants and fiber content of this diet (72). Furthermore, the findings of the current study showed an inverse association between the moderation component and levels of Gal-3. The moderation component indicated lower consumption of total cholesterol and saturated fatty acids, which are associated with higher inflammation (73).

This study has several strengths. The validity and reliability of the FFQ questionnaire have been well established (41). In addition, the analysis was adjusted for a variety of possible confounders.

However, this study also has several limitations. First, while the analysis was adjusted for the various cofounders, potentially residual effects might influence the outcome variables of interest. Second, this study included overweight and obese women; thus, the findings are not generalizable to the whole population. Future studies including men are needed to reveal if the link between DQI-I and inflammatory markers is gender-dependent. Third, the study design was cross-sectional. As a result, causality cannot be conferred due to the observational nature of this study. Future studies with prospective designs are needed. Finally, dietary data were collected using an FFQ questionnaire that is dependent on the subjects' memory, which might result in bias.

## Conclusion

The DQI-I probably has a negative association with hs-CRP in overweight and obese Iranian women. Furthermore, the higher score of adequacy probably has an inverse association with HOMA-IR and hs-CRP. In addition, the balance score probably has a negative association with TGF-β. The moderation score probably has a negative association with Gal-3, and the variety score probably has a negative association with HOMA-IR, MCP-1, and hs-CRP. The findings showed that the DQI-I might be a better prediction for HOMA-IR than other inflammatory markers. In summary, having higher DQI-I is probably associated with lower inflammatory markers in overweight and obese women. Future research with a larger sample size and both genders is needed to expand our knowledge regarding associations between diet quality indices and inflammatory markers in the Iranian population.

## Data availability statement

The raw data supporting the conclusions of this article will be made available by the authors, without undue reservation.

## Ethics statement

This study was conducted according to the guidelines laid down in the Declaration of Helsinki and all procedures involving human subjects were approved by the Ethics Commission of Tehran University of Medical Sciences (IR.TUMS.VCR.REC.1396.2615) and all participants signed written informed consent. The patients/participants provided their written informed consent to participate in this study.

## Author contributions

FS and KM implemented and designed the project. FS and SE analyzed and interpreted data. SN prepared the manuscript. SE, RB, SA-A, and AW revised the manuscript. KM supervised the overall project. All authors read and approved the final manuscript.
